# A Rare Case of Gangrenous Meckel’s Diverticulum and Acute Appendicitis Presenting As Closed-Loop Obstruction in a Young Adult

**DOI:** 10.7759/cureus.108627

**Published:** 2026-05-11

**Authors:** Chinmai Meka, Naveen Alexander, Bhuvaneshwari T. H.

**Affiliations:** 1 General Surgery, Sri Ramachandra Institute of Higher Education and Research, Chennai, IND

**Keywords:** appendicitis, bowel gangrene, closed loop obstruction, meckel's diverticulum, paracaecal hernia

## Abstract

Meckel’s diverticulum is the most common congenital anomaly of the gastrointestinal tract, arising from incomplete obliteration of the vitelline duct. Although often asymptomatic, it can present with life-threatening complications such as inflammation, bleeding, intestinal obstruction, and perforation. Closed-loop obstruction secondary to Meckel’s diverticulum is rare, and its coexistence with acute appendicitis is exceptionally uncommon, posing a diagnostic challenge. We report the case of a 22-year-old male who presented to the emergency department with a two-day history of diffuse abdominal pain and one day of bilious vomiting. Clinical examination revealed abdominal distension, diffuse tenderness, and bowel sounds that were sluggish, suggestive of intestinal obstruction. Contrast-enhanced computed tomography (CECT) of the abdomen and pelvis demonstrated clustering of small bowel loops medial to the caecum, raising suspicion for a paracaecal internal hernia with features of closed-loop obstruction involving mid-ileal loops, though bowel wall enhancement was preserved. Exploration revealed an inflamed appendix and a gangrenous Meckel’s diverticulum associated with interloop adhesions, resulting in a closed-loop obstruction of the adjacent ileal segment. Surgical management included resection of the gangrenous ileal segment along with the Meckel’s diverticulum, followed by a side-to-side ileo-ileal anastomosis using a linear stapler. Appendicectomy was performed concurrently. The postoperative course was uneventful, and the patient remained asymptomatic during a six-month follow-up period. This case highlights the rare coexistence of gangrenous Meckel’s diverticulum and acute appendicitis presenting as closed-loop obstruction, underscoring the importance of maintaining a high index of suspicion and a broad differential diagnosis in young patients presenting with an acute abdomen. Early surgical intervention remains crucial to prevent morbidity and ensure favourable outcomes.

## Introduction

Meckel’s diverticulum was initially documented by Fabricius Hildanus in 1598 and was later named after Johann Friedrich Meckel, who clarified its embryological basis in 1809 [1]. It represents a congenital abnormality arising from the failure of complete involution of the vitelline (omphalomesenteric) duct during fetal development. Although it is recognized as the most prevalent congenital anomaly of the gastrointestinal tract, affecting approximately 2% of the population, most individuals remain asymptomatic. Consequently, it is frequently discovered incidentally during surgical procedures, imaging studies, or postmortem examinations [2]. The classical “rule of 2s” is often cited: it occurs in 2% of individuals, measures about 2 inches in length, is typically located within 2 feet of the ileocecal valve, commonly presents before 2 years of age, and shows a male predominance with a 2:1 ratio [3].

From a clinical perspective, Meckel’s diverticulum can closely resemble acute appendicitis, as both conditions may present with right lower quadrant pain, nausea, and vomiting. Therefore, it should be included in the differential diagnosis of suspected appendicitis, especially when the appendix appears normal during surgery. In such situations, Meckel’s diverticulum is not infrequently identified during laparoscopic appendectomy. Among symptomatic cases, diverticulitis is the most common presentation, occurring in nearly 20% of patients. Other complications include bleeding, inflammation, perforation, and intestinal obstruction [4]. Inflammatory processes associated with Meckel’s diverticulitis can lead to the formation of adhesions between bowel loops, predisposing to small bowel obstruction and, in rare instances, closed-loop obstruction.

The vermiform appendix, another structure derived from the midgut, originates around the eighth week of gestation as a diverticular outgrowth from the cecum. As development progresses, it elongates into a narrow tubular structure while the cecum undergoes rotation and settles in the right iliac fossa. Acute appendicitis most commonly results from obstruction of the appendiceal lumen, which may be caused by fecaliths, lymphoid hyperplasia, neoplasms, ingested substances such as barium, or parasitic infestations including *Ascaris lumbricoides* and *Enterobius vermicularis*. Clinically, appendicitis may present in acute or chronic forms; the acute form evolves over hours to days, whereas chronic appendicitis may persist over a prolonged period. If not managed promptly, complications such as perforation, appendicular abscess, and generalized peritonitis may occur.

In this report, we describe a rare case of concurrent acute appendicitis and gangrenous Meckel’s diverticulum presenting as a closed-loop small bowel obstruction in a young adult with no prior surgical history. To the best of our knowledge, the coexistence of these two pathologies culminating in a closed-loop obstruction has not been previously reported in the literature. This case underscores the importance of maintaining a broad differential diagnosis in patients presenting with an acute abdomen and highlights the need for thorough intraoperative evaluation to identify multiple coexisting pathologies.

## Case presentation

A 22-year-old male presented to the emergency department with complaints of abdominal pain for two days and multiple episodes of bilious vomiting for one day. The abdominal pain was insidious in onset, intermittent, and gradually progressive in nature, with no aggravating or relieving factors. He also reported obstipation for one day. There was no history of fever, chills, diarrhea, or abdominal trauma. The patient had no known comorbidities and no prior history of abdominal surgery.

On examination, the patient was conscious, alert, and oriented to time, place, and person. His vital parameters were stable, with a blood pressure of 120/70 mmHg, heart rate of 100 beats per minute, and oxygen saturation of 99% on room air. Abdominal examination revealed a soft abdomen with tenderness localized to the right iliac fossa, right lumbar region, and hypogastric region. Guarding was noted in the right iliac fossa. Bowel sounds were sluggish, suggestive of evolving intestinal obstruction.

Initial laboratory investigations revealed leukocytosis and neutrophilia, while renal function tests and liver function tests were within normal limits. Arterial blood gas analysis was done, which was normal, indicating no significant metabolic derangement or tissue hypoperfusion at presentation, as shown in Table 1.

**Table 1:** Blood investigations showing leukocytosis and neutrophilia g: grams, dL: decilitre, cumm: cubic millimeter, mmol: millimole

S. No.	Test	Values	Units	Reference range
1	Hemoglobin	15.9	g/dL	12-17 g/dL
2	Total leukocyte count	16,900	lakhs/cumm	4000-11,000 lakhs/cumm
3	Neutrophils	85	%	45-70%
4	Serum sodium	140	mmol/litre	134-144 mmol/litre
5	Serum potassium	4.1	mmol/litre	3.5-5 mmol/litre
6	Serum chloride	92	mmol/litre	96-108 mmol/litre
7	Serum bicarbonate	23	mmol/litre	21-29 mmol/litre
8	pH	7.37	-	7.35-7.45
9	Serum lactate	0.75	mmol/litre	0.20-1.8 mmol/litre

**Figure 1:**
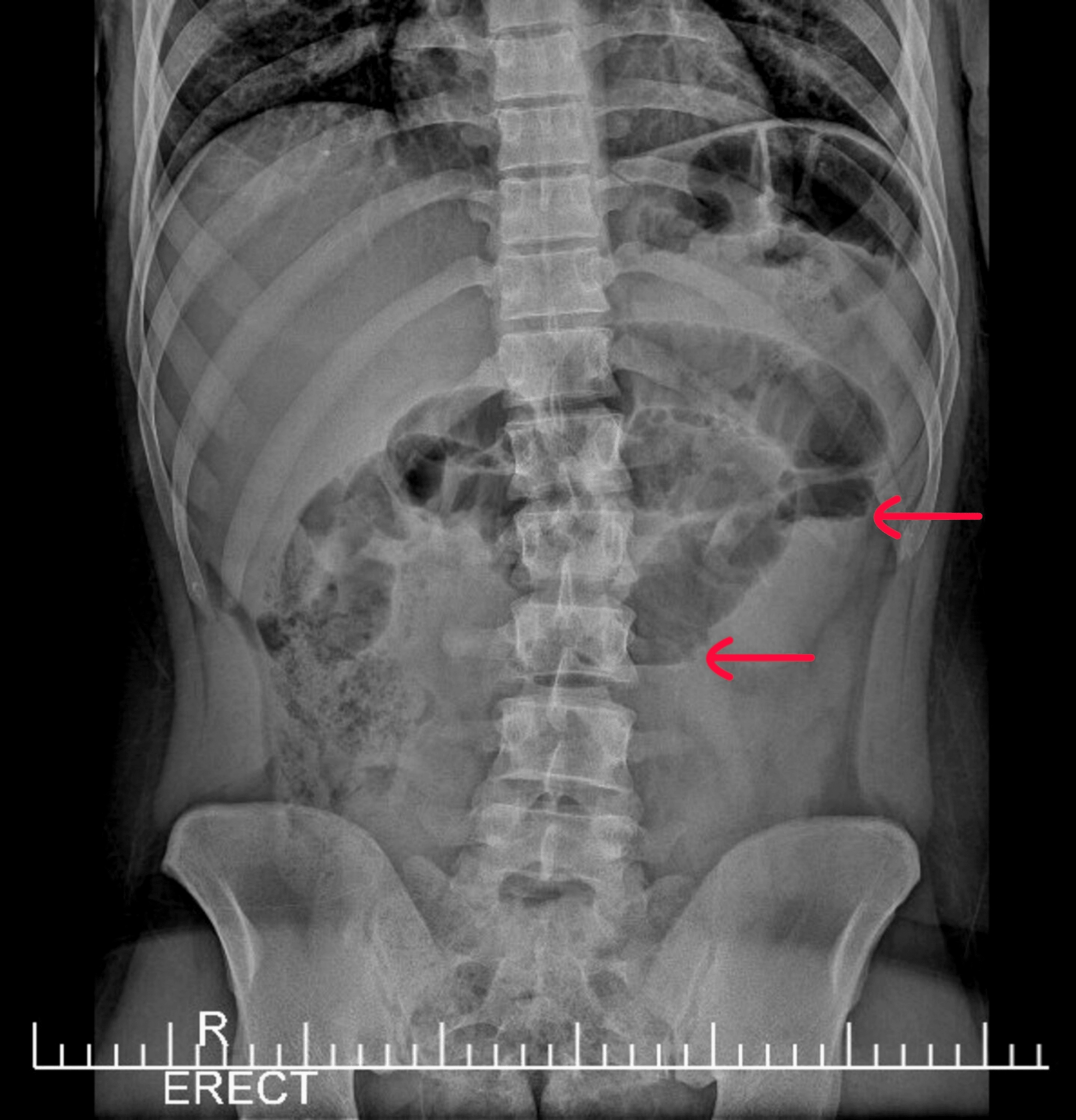
An erect X-ray abdomen showing multiple dilated bowel loops with air-fluid levels (red arrows)

An erect X-ray abdomen was done, which demonstrated multiple dilated bowel loops with air-fluid levels, consistent with intestinal obstruction (Figure 1).

**Figure 2:**
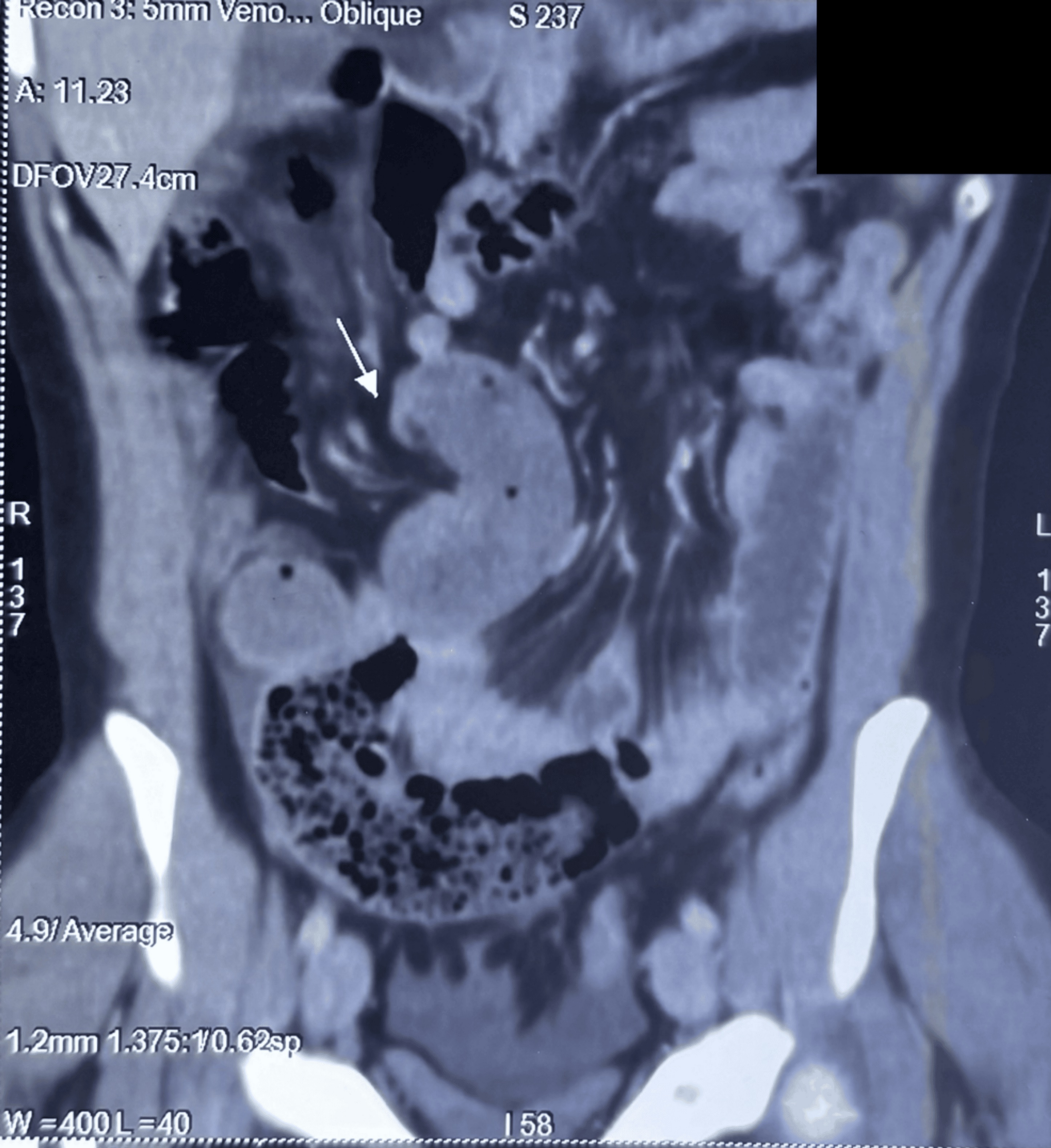
Coronal section of contrast-enhanced computed tomography (CECT) abdomen and pelvis showing dilated small bowel segment (white arrow) indicating closed-loop obstruction

Contrast-enhanced computed tomography (CECT) of the abdomen and pelvis was done, revealing clustering of small bowel loops medial to the caecum, suggestive of a paracaecal internal hernia with features of a closed-loop obstruction involving mid-ileal loops, with preserved bowel wall enhancement (Figure 2).

**Figure 3:**
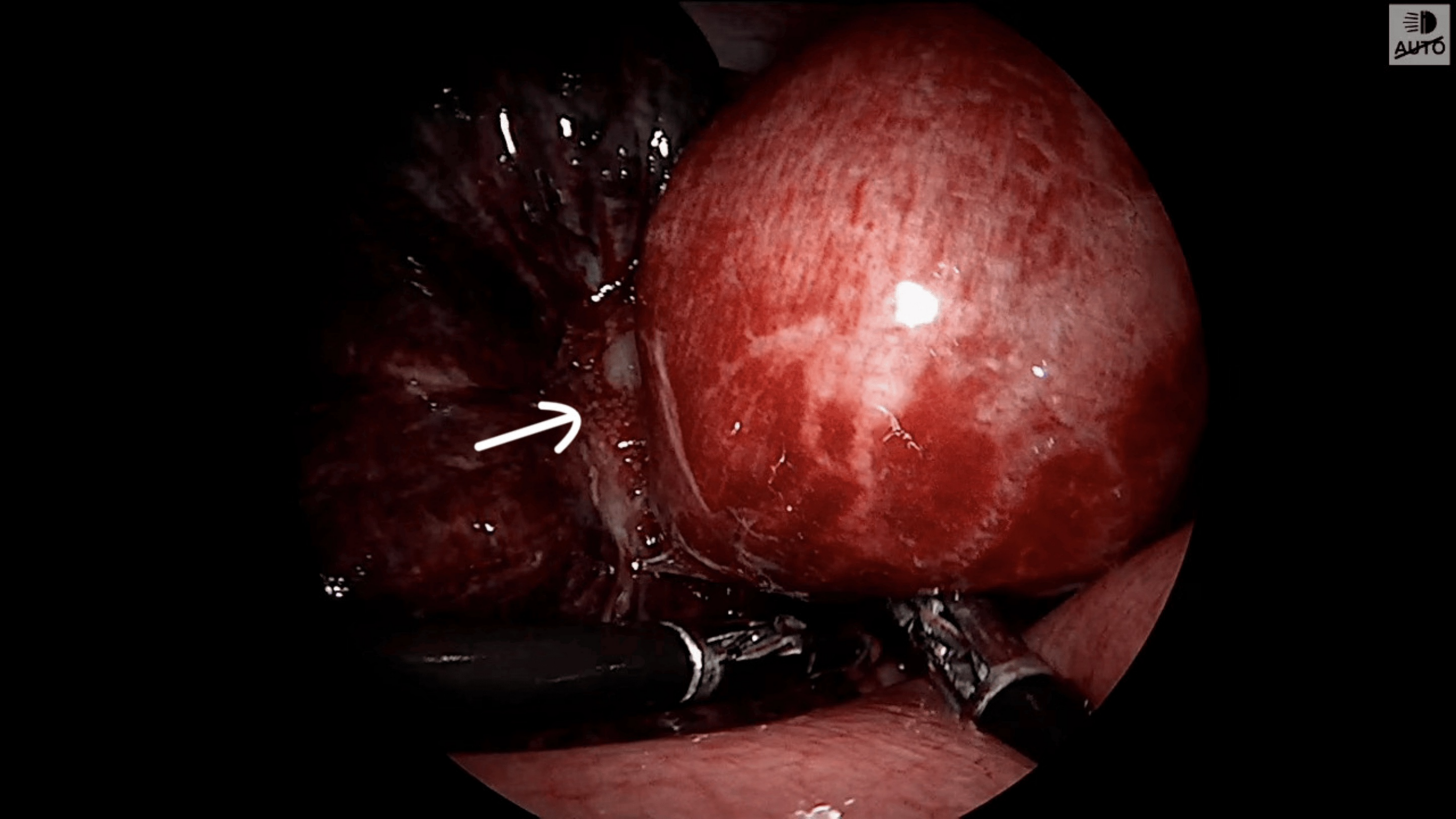
Laparoscopic image showing gangrenous segments of small bowel with dense interloop adhesions (white arrow)

Given the clinical and radiological findings, the patient was taken up for emergency diagnostic laparoscopy. Intraoperatively, gangrenous segments of small bowel with dense interloop adhesions (Figure 3) causing a closed-loop obstruction were identified.

**Figure 4:**
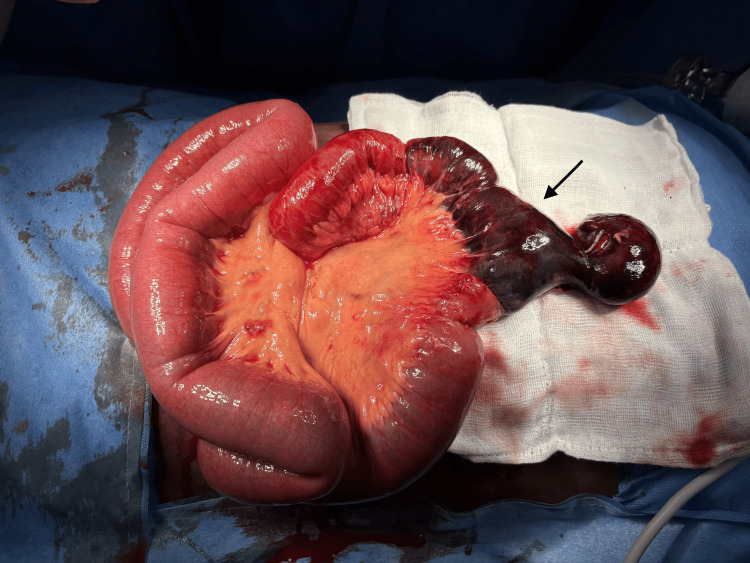
Intraoperative image of a gangrenous segment of ileum along with Meckel’s diverticulum (black arrow)

Due to the extent of bowel involvement and technical difficulty in performing adhesiolysis laparoscopically, the procedure was converted to an exploratory laparotomy via a midline incision. Intraoperative findings included a gangrenous segment of ileum associated with a Meckel’s diverticulum (Figure 4).

**Figure 5:**
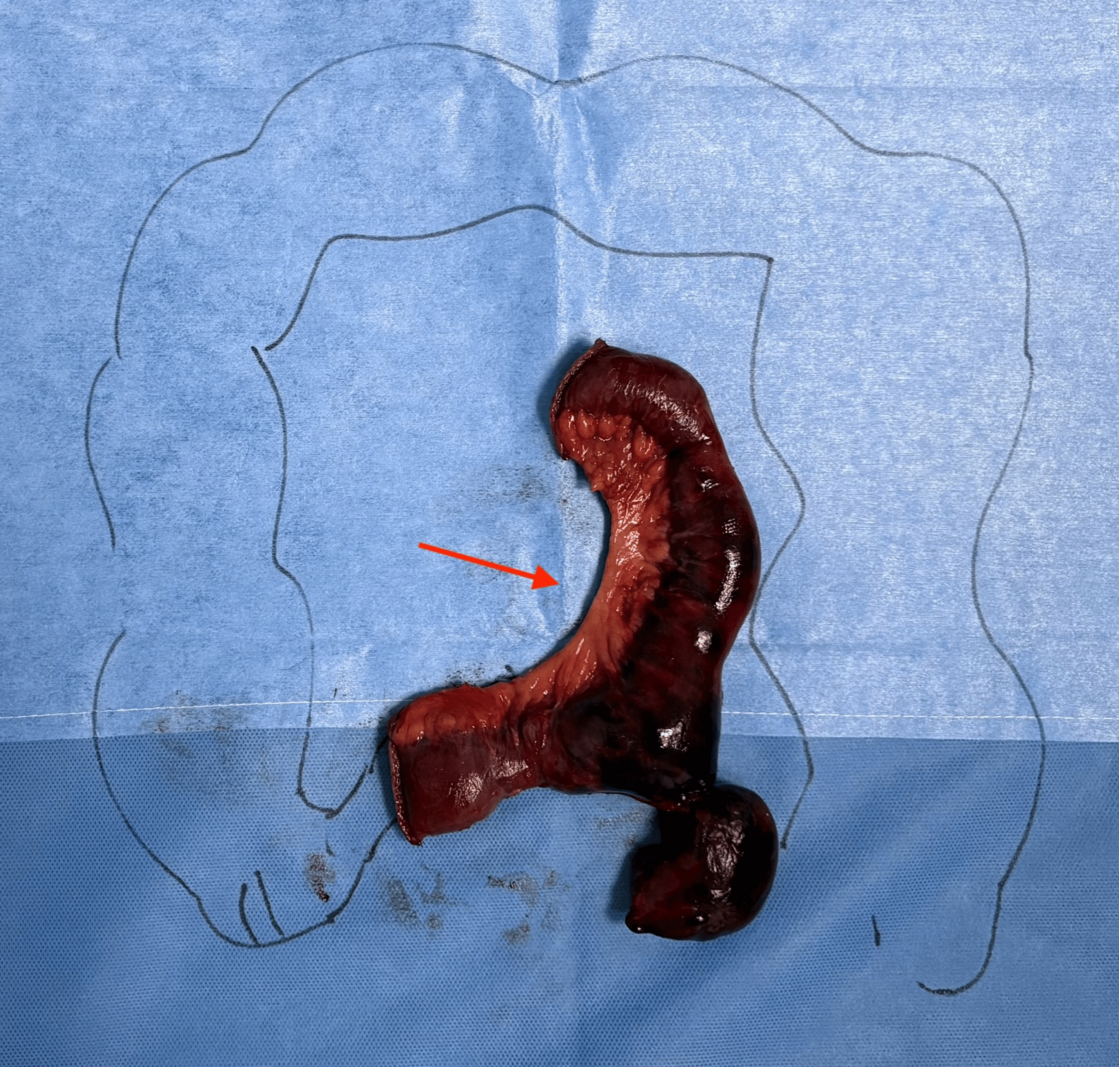
Specimen image showing gangrenous ileal segment extending approximately 15 cm proximal to the diverticulum and up to 5 cm distally (red arrow)

The gangrene extended approximately 15 cm proximal to the diverticulum and up to 5 cm distally (Figure 5).

The proximal ileal loops were markedly dilated, while the distal bowel loops were collapsed, confirming the presence of a closed-loop obstruction. Additionally, an inflamed appendix consistent with acute appendicitis was noted. Surgical management involved resection of the gangrenous ileal segment along with the Meckel’s diverticulum, followed by a side-to-side ileo-ileal anastomosis using a linear stapler. Appendicectomy was performed concurrently. Two intra-abdominal drains were placed - one in the pelvis and another near the anastomotic site.

Postoperatively, the patient was managed in the surgical ward with nil per os (NPO) status, intravenous fluids, antibiotics, and close monitoring, including strict input-output and abdominal girth charting. On postoperative day (POD) 3, the patient passed flatus, following which the Ryle’s tube was removed and oral liquids were initiated, which he tolerated well. By POD 4, the patient was advanced to a soft diet. On POD 5, the drain output was minimal, and both drains were removed. The patient remained clinically stable, tolerated a normal diet, and had no abdominal complaints; hence, he was discharged. Histopathological examination confirmed gangrenous small bowel associated with Meckel’s diverticulum and features consistent with acute appendicitis.

## Discussion

We report a rare and clinically challenging case involving the simultaneous occurrence of three surgical pathologies - gangrenous Meckel’s diverticulum, acute appendicitis, and closed-loop small bowel obstruction - in a young adult. While acute appendicitis remains one of the most common causes of acute abdomen, this case underscores the importance of maintaining a broad differential diagnosis, particularly in atypical or inconclusive presentations. Meckel’s diverticulum, though often asymptomatic, should always be considered as a potential differential in patients presenting with right lower quadrant pain suggestive of appendicitis. Internal hernias account for up to 6% of the patients with intestinal obstruction [5], and their complications frequently necessitate urgent surgical intervention, carrying considerable morbidity and mortality.

A key surgical principle highlighted by this case is the necessity for thorough intraoperative evaluation. In situations where the appendix appears normal during surgery despite clinical suspicion of appendicitis, further exploration of the distal ileum is warranted to identify alternative pathologies such as Meckel’s diverticulum. However, as demonstrated in this patient, the coexistence of multiple pathologies can further complicate the clinical picture, and surgeons must remain vigilant for concurrent disease processes rather than attributing symptoms to a single diagnosis.

The clinical manifestations of Meckel’s diverticulum are highly variable. A comprehensive review by Hansen and Søreide reported that intestinal obstruction is the most common presentation of symptomatic Meckel’s diverticulum, followed by gastrointestinal bleeding and diverticulitis [6]. Interestingly, more than half of symptomatic cases occur in children under 10 years of age, making adult presentations relatively uncommon and often diagnostically challenging. The mechanism of obstruction may include volvulus around fibrous bands, intussusception, Littre’s hernia, or, as in our case, interloop adhesions secondary to inflammation leading to closed-loop obstruction and subsequent bowel ischemia [7].

Radiological diagnosis of Meckel’s diverticulum remains difficult. As noted by Shademan and Tappouni, Meckel’s diverticulum can be misinterpreted as other causes of acute abdomen, including appendicitis or small bowel obstruction, on cross-sectional imaging [8]. In our case, preoperative contrast-enhanced CT suggested a paracaecal internal hernia with closed-loop obstruction, without clear identification of the underlying Meckel’s diverticulum. This highlights the limitations of imaging in distinguishing Meckel’s diverticulum from other intra-abdominal pathologies, particularly in the presence of complications such as inflammation or ischemia. Furthermore, Meckel’s diverticulum may contain ectopic mucosa, most commonly gastric or pancreatic tissue, which can lead to mucosal ulceration and bleeding, further contributing to its varied clinical presentation [9].

Surgical management of Meckel’s diverticulum depends on the clinical scenario and intraoperative findings. Both laparoscopic and open approaches are described in the literature. Options include simple diverticulectomy or segmental ileal resection with primary anastomosis, particularly in cases complicated by inflammation, perforation, or gangrene. In our patient, initial diagnostic laparoscopy revealed gangrenous small bowel with dense interloop adhesions causing a closed-loop obstruction. Due to the extent of bowel involvement and technical difficulty in laparoscopic adhesiolysis, conversion to an open approach was necessary. Exploratory laparotomy revealed a gangrenous ileal segment associated with Meckel’s diverticulum, extending proximally and distally, necessitating segmental resection and ileo-ileal anastomosis. Concurrent appendicectomy was performed in view of intraoperative evidence of acute appendicitis. Histopathological examination confirmed the presence of gangrenous small bowel with Meckel’s diverticulum and acute appendicitis, validating the intraoperative findings.

This case is unique due to the rare coexistence of gangrenous Meckel’s diverticulum and acute appendicitis presenting as a closed-loop obstruction, a combination that, to the best of our knowledge, has not been previously reported in the literature. It highlights several important clinical lessons: the diagnostic limitations of imaging in complex abdominal presentations, the importance of considering multiple concurrent pathologies, and the need for prompt surgical intervention in cases of suspected bowel ischemia.

## Conclusions

We report a rare case of the simultaneous occurrence of acute appendicitis, gangrenous Meckel’s diverticulum, and closed-loop small bowel obstruction in a young adult. Although each of these entities can independently present as an acute abdomen, their coexistence is exceedingly uncommon and presents a significant diagnostic challenge. This case highlights the limitations of preoperative imaging, as the initial radiological findings suggested a paracaecal hernia, while the definitive diagnosis was established only through surgical exploration. Prompt operative intervention, including timely conversion from laparoscopy to laparotomy, enabled appropriate management with resection of the gangrenous ileal segment containing Meckel’s diverticulum, ileo-ileal anastomosis, and appendicectomy, resulting in a favorable outcome. This case underscores the importance of maintaining a high index of suspicion for less common pathologies such as complicated Meckel’s diverticulum in patients presenting with features suggestive of acute appendicitis or intestinal obstruction. It also emphasizes that multiple pathological processes may coexist and contribute to the clinical presentation, necessitating a thorough intraoperative evaluation rather than reliance on a single diagnosis. Early recognition and decisive surgical management remain critical in reducing morbidity and improving outcomes in such complex presentations of acute abdomen.
